# An advanced approach to identify antimicrobial peptides and their function types for penaeus through machine learning strategies

**DOI:** 10.1186/s12859-019-2766-9

**Published:** 2019-06-10

**Authors:** Yuan Lin, Yinyin Cai, Juan Liu, Chen Lin, Xiangrong Liu

**Affiliations:** 1 Department of Computer Science, School of Information Science and Technology, Xiamen University, Xiamen, 361005 China; 2 Sparebanken Vest, Jonsvollsgaten 2, 5011 Bergen, Bergen, 5058 Norway; 3 Department of Instrumental and Electrical Engineering, School of Aerospace Engineering, Xiamen University, Xiamen, 361005 China

**Keywords:** Antimicrobial peptides, Feature extraction, Multi-label classification, Machine learning

## Abstract

**Background:**

Antimicrobial peptides (AMPs) are essential components of the innate immune system and can protect the host from various pathogenic bacteria. The marine environment is known to be one of the richest sources for AMPs. Effective usage of AMPs and their derivatives can greatly improve the immunity and breeding survival rate of aquatic products. It is highly desirable to develop computational tools for rapidly and accurately identifying AMPs and their functional types, for the purpose of helping design new and more effective antimicrobial agents.

**Results:**

In this study, we made an attempt to develop an advanced machine learning based computational approach, MAMPs-Pred, for identification of AMPs and its function types. Initially, SVM-prot 188-D features were extracted that were subsequently used as input to a two-layer multi-label classifier. In specific, the first layer is to identify whether it is an AMP by applying RF classifier, and the second layer addresses the multi-type problem by identifying the activites or function types of AMPs by applying PS-RF and LC-RF classifiers. To benchmark the methods,the MAMPs-Pred method is also compared with existing best-performing methods in literature and has shown an improved identification accuracy.

**Conclusions:**

The results reported in this study indicate that the MAMP-Pred method achieves high performance for identifying AMPs and its functional types.The proposed approach is believed to supplement the tools and techniques that have been developed in the past for predicting AMPs and their function types.

## Background

Antimicrobial peptides (AMPs) are crucial components of the innate immune system and can protect the host from various pathogenic bacteria and viruses. They are generally short peptides with 10–50 amino acids [1] and have very low sequence homology to one another. AMPs nowadays have attracted increased attention of research owing to their broad-spectrum antimicrobial activity and more importantly to the fact that AMPs may overcome the antimicrobial resistance, which makes it a potential alternative therapeutic agent for humans or a substitute to conventional antibiotics.

However, the mechanisms of action of AMPs, as well as their structure-activity relationships, are not completely understood [2]. Identification and optimization of AMPs can provide a theoretical basis for discovery and design of new and more effective antimicrobial agents. For instance, a multidimensional signature model was proposed in [3] that facilitates discovery of AMPs and offers insights into the evolution of molecular determinants. Experimental and computational studies are generally devoted to dealing with this challenging task. Computational methods were developed to accelerate the process of prediction and classification of AMPs. Recently, approaches based on machine learning techniques are commonly adopted due to their high efficiency, high speed, low cost and generalization abilities. They can sufficiently mine the intrinsic linear and non-linear relationship between antibacterial activity and biochemical attributes, which is suitable for dealing with large scale antimicrobial peptide prediction tasks with complex models.

Methods of choice include support vector machine (SVM) [4–7], nearest neighbor [8] or k-nearest neighbor algorithm [9], random forests (RFs) [10]), decision tree model [11], hidden Markov models (HMMs) [12], and neural network model [13] which seek for prediction power in a context of supervised classification. Most recent work includes a "deep" network architecture for chemical data analysis and classification together with a prospective proof-of-concept application proposed in [14]. Some predictors only apply binary classifiers to identify whether a query peptide sequence is AMP or not, such as [4, 5, 8]. Multi-class classifiers have also been developed which obtained more detailed quantitative results. Lira et al. [11] created a decision tree model to classify the antimicrobial activities of synthetic peptides into four classes. ClassAMP [4] has been developed to predict the propensity of a peptide sequence to have antibacterial, antifungal, or antiviral activity. However, it can be seen by a comparison of the sequences in APD database [15, 16] that a same sequence may occur in different subclasses, which in fact a very common phenomenon. Therefore, it is highly desirable to develop mechanisms for rapidly and accurately learning from multi-label datasets, for the purpose of helping design new and more effective antimicrobial agents. Considering various possible functional types of AMPs, Xiao et al. proposed a two-level multi-label classifier iAMP-2L, where an improved fuzzy K-nearest neighbour (FKNN) algorithm was applied, and after the AMPs are first identified, the positive samples are subjected to regular multi-label learning processing [9]. The prediction accuracy for 4 types of AMPs was further improved in [17]. Zhou’s method [18] has applied the LIFT multi-label learning algorithm to predict 5 types of AMPs and achieved 70% accuracy of prediction.

This paper aims to develop an advanced method, MAMPs-Pred, for classification and prediction of AMPs and their function types, which proves to achieve an improved prediction accuracy upon state of the art mechanisms. The marine environment is known to be one of the richest sources for AMPs. It is meaningful to predict the AMPs and their function types of penaeus by this method, which has helped us to understand the immune system of marine species. In addition, it eases subsequent mining and exploration of antimicrobial activity of other species.

In this approach, a 188-D feature set constructed from SVM-Prot features [19, 20] were used to map the peptide sequences to numeric feature vectors, which were subsequently used as input to a two-layer multi-label classifier. The first layer is to identify whether a query peptides sequence is an AMP, and the second layer addresses the multi-type problem by identifying whether an AMP belongs to multiple function types. Different classification methods were compared, and the results were discussed and analyzed. In short, a combination of first-layer 188D-RF classifier and second-layer PS-RF or LC-RF classifier is proved to have achieved the best performance. The proposed approach achieved higher accuracy than existing approaches of best performance, while performed upon benchmark dataset. In addition, the quality of the prediction was verified when applied to penaeus sequences. The proposed method may play an important complementary role to the existing predictors in this area.

## Materials and methods

### Benchmark dataset

For the convenience of later description, the benchmark dataset is expressed by

1 $$\begin{array}{*{20}l} s &= s^{AMPs} \cup s^{non-AMPs} \end{array} $$

Where *s*^*AMPs*^ is the AMPs dataset consisting of AMPs sequences only, *s*^*non*−*AMPs*^ the non-AMP dataset with non-AMP sequences only, and ∪ is the symbol for union in the set theory. The peptide sequences in *s*^*AMPs*^ were fetched from the APD database [15, 16], which has collected all antimicrobial peptides from the PubMed, PDB, Google and Swiss-Prot databases. According to their different functional types, the AMP sequences can be further classified into 16 categories; i.e.,

2 $$\begin{array}{*{20}l} s &= s_{1}^{AMPs} \cup s_{2}^{AMPs} \cup s_{3}^{AMPs} \cup \ldots \cup s_{16}^{AMPs} \end{array} $$

Where the subscripts 1, 2, 3,...,16 represent “Wound healing”, “Spermicidal”, “Insecticidal”, “Chemotactic”, “Antifungal”, “Anti-protist”, “Antioxidant”, “Antibacterial”, “Antibiotic”, “Antimalarial”, “Antiparasital”, “Antiviral”, “Anticancer/tumor”, “Anti-HIV”, “Proteinase inhibitor” and “Surface immobilized”. The lengths of AMPs are varying within the region from 5 to 100 amino acids. Note that among the original 2954 *s*^*AMPs*^ sequences, 278 sequences have unknown antibacterial activity.

Furthermore, to reduce homology bias and redundancy, the program CD-HIT [21] was utilized to winnow those sequences that have ≥ pairwise sequence identity to any other in a same subset. The alignment bandwidth of the CD-HIT field is set to 5 according to the shortest length of AMPs. To ensure that each subset has enough samples for statistic processing, and to ensure that all categories are covered, the CD-HIT only performs redundancy removal to a subset of samples with sequence numbers larger than 180, which means that the de-redundancy processing are only performed for antifungal, antibacterial, antiviral and anti-cancer polypeptides. Finally, we obtained 2618 AMPs as the current benchmark dataset *s*^*AMPs*^ as shown in Table 1.

**Table 1:** Preprocessed benchmark dataset

Function	Dataset	Function type	Sequence
AMPs	$s_{1}^{AMPs}$	Wound healing	18
	$s_{2}^{AMPs}$	Spermicidal	13
	$s_{3}^{AMPs}$	Insecticidal	28
	$s_{4}^{AMPs}$	Chemotactic	57
	$s_{5}^{AMPs}$	Antifungal	593
	$s_{6}^{AMPs}$	Anti-protist	4
	$s_{7}^{AMPs}$	Antioxidant	22
	$s_{8}^{AMPs}$	Antibacterial	1297
	$s_{9}^{AMPs}$	Antibiotic	32
	$s_{10}^{AMPs}$	Antimalarial	25
	$s_{11}^{AMPs}$	Antiparasital	101
	$s_{12}^{AMPs}$	Antiviral	125
	$s_{13}^{AMPs}$	Anticancer	125
	$s_{14}^{AMPs}$	Anti-HIV	109
	$s_{15}^{AMPs}$	Proteinase inhibitor	26
	$s_{16}^{AMPs}$	Surface immobilized	43
	*s* ^*AMPs*^		2618
non-AMPs	*s* ^*non*− *AMPs*^		4371

The negative samples *s*^*non*−*AMPs*^ contains polypeptide sequences *s*^*non*−*AMPs*−*Pept*^, and protein fragments *s*^*non*−*AMPs*−*Prot*^.

Where *s*^*non*−*AMPs*−*Pept*^ were constructed according to following procedures:

1. Collected all the polypeptide sequences *s*^*UNP*−*Peptide*^ with length 1 to 15483, in total 79378, from the UniProt database.
2. Removed any sequence that already exists in *s*^*AMPs*^, any sequence that contains any code other than the 20 native amino acid codes, and any sequence with length less than 5 or larger than 100.
3. The process is described by following equation, and at this point 10503 sequences *s*^*non*−*AMPs*−*Pept*^ were obtained.
  3 $$\begin{array}{*{20}l} s^{non-AMPs-Pept}&=s^{UNP-Peptide}-s^{AMPs}-seq_{illeg}\\ (len \in [5,100]) \end{array} $$

On the other hand, *s*^*non*−*AMPs*−*Prot*^ were constructed according to following procedures:

1. Obtained Pfam families that *s*^*AMPs*^ belong to. Because some AMPs are homologous and have the same family number, we remove duplicate family numbers from Pfam and get de-redundant families posPfam.
2. Removed posPfam from the Pfam families and obtained negPfam. Fetched a random protein sequence with the length between 5 and 100 from each negPfam family.
3. The process is described by following equation. In total 109 short protein sequences *s*^*non*−*AMPs*−*Prot*^ were obtained.
  4 $$\begin{array}{*{20}l} s^{non-AMPs-Prot}&=Ran(Pfam-posPfam)\\ (len \in [5,100]\!) \end{array} $$

The *s*^*non*−*AMPs*^ were constructed by following equation.

5 $$\begin{array}{*{20}l} s^{non-AMPs}&=s^{non-AMPs-Pept} \cup s^{non-AMPs-Prot} \end{array} $$

The CD-HIT [21] program was then applied to winnow *s*^*non*−*AMPs*^. Finally, 4371 sequences were constructed, which were used to form the negative samples dataset *s*^*non*−*AMPs*^ as shown in Table 1.

### Feature extraction

In machine learning, choosing informative, discriminating and independent features is a crucial step for the success of a prediction method. The optimal feature set shall be able to capture the distribution patterns of the dataset.

In this study, we have adopted two feature extraction algorithms for comparison, which are SVM-Prot 188-D based on 8 types of physical-chemical properties and amino acid composition, and Pseudo amino acid composition features (Co-Pse-AAC) based on 5 types of physical-chemical properties respectively.

SVM-Prot is a web server for protein classification. It constructs 188-D features for protein sequences description and classification [19, 20]. The features have been applied successfully in several protein identification works, such as cytokines [22, 23] and enzymes [24, 25]. The extracted features include hydrophobicity, normalized van der Waals volume, polarity, polarizability, charge, surface tension, secondary structure and solvent accessibility [19]. For each of these 8 types of physical-chemical properties, some feature groups were designed to describe global information of protein sequences. These feature groups contain composition (C), transition (T) and distribution (D) [19, 26]. Thus, the dimension of each feature vector is 21. In addition, considering amino acid composition (AAC), the protein structure is composed of 20 amino acids. The dimension of 188-D features is therefore expressed as below formula:

6 $$\begin{array}{*{20}l} D_{188-D}&=\sum_{i=1}^{L} D_{21Vct} + D_{aac} \end{array} $$

Where L is the number of features, which is 8 in this context. Take Cecropin A as an example. The 188-D features of Cecropin A is showed in Table 2. To the best of our knowledge, it is the first attempt in literature to apply SVM-Prot 188-D feature set composition in AMPs and non-AMPs classification and identification.

**Table 2:** 188-D feature of cecropin A

Sequence	KWKLFKKIEKVGQNIRDGIIKAGPAVAVVGQATQIAK						
Property	Value of feature vector						
Amino acid composition	13.5	0.0	2.70	2.70	2.70	10.8	0.0
	135	00	27.0	27.0	27.0	108	00
	13.5	18.9	2.70	0.00	2.70	2.70	8.10
	135	189	27.0	0.00	27.0	27.0	81.0
	2.70	0.00	2.70	10.8	2.70	0.00	
	27.0	00	27.0	108	27.0	00	
Hydro-phobic	37.8	29.7	32.4	19.4	30.5	19.4	2.70
	378	297	324	444	555	444	27
	16.2	35.1	45.9	100.	32.4	48.6	64.8
	162	351	459	000	324	486	648
	81.0	97.2	5.40	13.5	40.5	70.2	94.5
	810	972	54	135	405	702	945

On the other hand, Pseudo amino acid composition features (Co-Pse-AAC) [27] as an efficient computation tool has been diffusely leveraged for protein sequences in predicting protein structures and functions, as well as DNA and RNA sequences [28]. The 40-dimension Co-Pse-AAC features were extracted and sufficiently incorporate the effects of sequence order. This method has taken 5 types of physical-chemical properties into consideration.

### Data balancing

Most machine learning classification algorithms are sensitive to the imbalanced data sets [29]. The classifiers tend to have a higher recognition rate for the majority class, which makes it difficult to identify the minority class correctly [30–32]. In this study, there were 2718 AMPs samples and 4371 non-AMPs samples, which were highly imbalanced. In order to eliminate the over fitting problem caused by imbalanced data, we have applied two sampling mechanisms to construct the training dataset.

Firstly, we have implemented a random-under-sampling method to down sample the large class set *s*^*non*−*AMPs*^, so that the sample number of large class set equals the small class set, and the resulting training dataset is defined as *s*_*train*_. Another method we have applied is weighted random sampling [33], which has balanced the dataset by applying different weights to the unbalanced samples. Given that the ratio of *s*^*AMPs*^ and *s*^*non*−*AMPs*^ is approximately equal to 3:5, weight factor 5 and 3 were applied to *s*^*AMPs*^ and *s*^*non*−*AMPs*^ respectively, and the obtained train dataset is defined as *s*_*weight*−*tr*_.

### Test dataset

The test dataset was constructed by following method. Firstly we randomly pick up 1382 negative samples from the sequences that have been deleted from *s*^*non*−*AMPs*^ in the CD-HIT process, and noted it by *s*^*non*−*AMPs*−*DEL*^. Further, in the phrase of acquiring benchmark dataset from APD (The Antimicrobial Peptide Database) database, there are 278 sequences with unknown antibacterial activity among the original 2954 *s*^*AMPs*^ sequences, which is defined by *s*^*non*−*AMPs*−*NOACT*^.

The 278 *s*^*non*−*AMPs*−*NOACT*^ sequences, together with the 1382 *s*^*non*−*AMPs*−*DEL*^, form the independent test dataset *S*_*test*_ for the first layer of our two-layer multi-label classifier, which is in total 1660 samples.

The 278 *s*^*non*−*AMPs*−*NOACT*^ sequences were also applied as prediction dataset for the second layer of our two-layer multi-label classifier, which will be illustrated in following chapters.

### Two-layer multi-label classifier

In machine learning, multi-label classification is the problem of finding a model that maps inputs x to binary vectors y, i.e., assigning a value of 0 or 1 for each label in y. In the multi-label problem there is no constraint on how many of the classes the instance can be assigned to. An overview of multi-label classification is available at [34].

In general, the methods to study multi-label classification can be divided into two categories: adapted algorithm methods and problem transformation methods. Some classification models have been adapted to the multi-label task, without requiring problem transformations. For instance, AdaBoost.MH and AdaBoost.MR are extended versions of AdaBoost for multi-label data. And the ML-kNN algorithm extends the k-NN classifier to multi-label data. Examples also include decision trees, neural networks adapted for multi-label learning.

Problem transformation methods fall into another category of multi-label classification. With converting multi-label problems into one or more single-label problems, literally existing single-label classifier can be used to meet the multi-label classification requirements. Representative algorithms include Binary Relevance (BR), Classifier Chains (CC), Label Combination Method (LC/LP), Integrated LP Method Rakel, and Pruned Sets Method (PS). BR amounts to independently training one binary classifier for each label; CC is similar to BR, except that it takes into account label dependencies; LC/LP treats each label combination as a new label and implicitly considers the label.

A polypeptide can be a non-AMP that does not have any antimicrobial activity. It is actually a prediction problem with negative samples, which cannot be handled directly by traditional multi-label classification. Incorporating non-AMPs rationally into predictive models is an essential issue for multi-label classification to predict function types of AMPs. To address this issue, we improve upon the state of the art in multi-label classification and make several contributions.

For the first-layer classifier in identifying a query peptide sequence as an AMP or non-AMP, the random forest (RF) algorithm was applied as a base classifier because of its good performance and simple-to-use feature. Random forest is an ensemble method in which a classifier is constructed by combining several independent base classifiers. The individual predictions are aggregated to combine into a final prediction, based on a majority voting on the individual predictions. By averaging several trees, there is a significantly lower risk of over fitting.

For the second layer classifier in identifying which functional type(s) the query AMP peptide sequence belongs to, a task of multi-label classification was launched. We choose Meka/Mulan open source framework to implement our second layer multi-label classifier. Meka is based on the Weka machine learning toolkit, one of the well-known data mining platforms (http://www.cs.waikato.ac.nz/ml/weka/), and integrates the open-source Java library Mulan framework for providing the capability of multi-label datasets learning. Meka proposed a trimming set method and a Classifier Chains (CC) method, and uses logarithmic loss to punish misplaced tags to prevent partial misprediction in the overall label distortion. For the second-layer prediction, PS-RF or LC-RF is applied as a base multi-label classifier due to its performance.

### Measurement metrics

The metrics Sensitivity (SN), specificity (SP), overall accuracy (Acc) and Matthew’s correlation coefficient (Mcc) were applied to measure the performance of the first-layer classifier [18, 35–40], where *TP*_*i*_,*FP*_*i*_,*TN*_*i*_,*FN*_*i*_ denote the numbers of true positive instances, false positive instances, true negative instances and false negative instances respectively.

7 $$\begin{array}{*{20}l} SN &= \frac{TP_{i}}{TP_{i} + {FN}_{i}} \end{array} $$

8 $$\begin{array}{*{20}l} SP &= \frac{TN_{i}}{FP_{i} + {TN}_{i}} \end{array} $$

9 $$\begin{array}{*{20}l} Acc &= \frac{TP_{i} + {TN}_{i}}{TP_{i} + {FP}_{i} + {TN}_{i} +{FN}_{i}} \end{array} $$

10 $$ {\begin{aligned} Mcc &= \frac{TP_{i} \times {TN}_{i} - {FP}_{i} \times {FN}_{i}} {\sqrt{({TP}_{i} + {FP}_{i}) \times ({TN}_{i} + {FN}_{i}) \times ({TP}_{i} + {FN}_{i}) \times ({TN}_{i} + {FP}_{i})}} \end{aligned}}  $$

The metric Exact-Match Ratio (EMR), Hamming-Loss (H-Loss), Accuracy (Acc), Precision (Precison, Recall), Ranking-Loss (RL), Log-Loss, One-error (OE), F1-Measure (F1-Mic, F1-Mac) were applied for evaluation the second-layer multi-label classifier.

11 $$\begin{array}{*{20}l} EMR(\Lambda_{t}) &= \frac{1}{K} \sum_{i=1}^{K} (\tilde{y_{i}} = y_{i}) \end{array} $$

12 $$\begin{array}{*{20}l} H-Loss(\Lambda_{t}) &= \frac{1}{KL} \sum_{i=1}^{K} \frac{|\tilde{y_{i}} \cup {y_{i}}|-|\tilde{y_{i}} \cap {y_{i}}|}{L} \end{array} $$

13 $$\begin{array}{*{20}l} Acc(\Lambda_{t}) &= \frac{1}{K} \sum_{i=1}^{K} \frac{|\tilde{y_{i}} \cup {y_{i}}|}{|\tilde{y_{i}} \cap {y_{i}}|} \end{array} $$

14 $$\begin{array}{*{20}l} Precision(\Lambda_{t}) &= \frac{1}{K} \sum_{i=1}^{K} \frac{|\tilde{y_{i}} \cap {y_{i}}|}{\tilde{y_{i}}} \end{array} $$

15 $$\begin{array}{*{20}l} Recall(\Lambda_{t}) &= \frac{1}{K} \sum_{i=1}^{K} \frac{|\tilde{y_{i}} \cap {y_{i}}|}{y_{i}} \end{array} $$

16 $$\begin{array}{*{20}l} F1(\Lambda_{t}) &= \frac{2.0 \times Precision(\Lambda_{t}) \times Recall(\Lambda_{t})}{Precision(\Lambda_{t}) + Recall(\Lambda_{t})}  \\ OE(\Lambda_{t}) &= \frac{1}{K} \sum_{i=1}^{K} \{[{argmax}_{y \in Y} h(x_{i}, y)] \not\in y_{i}\}  \\ &= \frac{1}{K} \sum_{i=1}^{K} \frac{2 |\tilde{y_{i}} \cap {y_{i}}|}{|\tilde{y_{i}}| + |y_{i}|} \end{array} $$

17 $$\begin{array}{*{20}l} RL(\Lambda_{t}) &\!\!=\frac{1}{K} \sum_{i=1}^{K} \frac{1}{|\tilde{y_{i}}| \!\times\! |y_{i}|} |\{ (y_{1}, y_{2}) | f_{t} h((x_{i}, y_{1}))  \\ \leq f_{t} h((x_{i}, y_{2})) \}| \end{array} $$

18 $$\begin{array}{*{20}l} Log-Loss(\Lambda_{t}) &= \frac{1}{KL} \sum_{i=1}^{K} \sum_{j=1}^{L}\\ & \left\{min\left[-Log-Loss\left(\tilde{w_{j}^{i}}, y_{j}^{i}\right), ln(K)\right]\right\} \end{array} $$

## Results

### First classifier - Identifying AMPs or non-AMPs

**Fig. 1:**
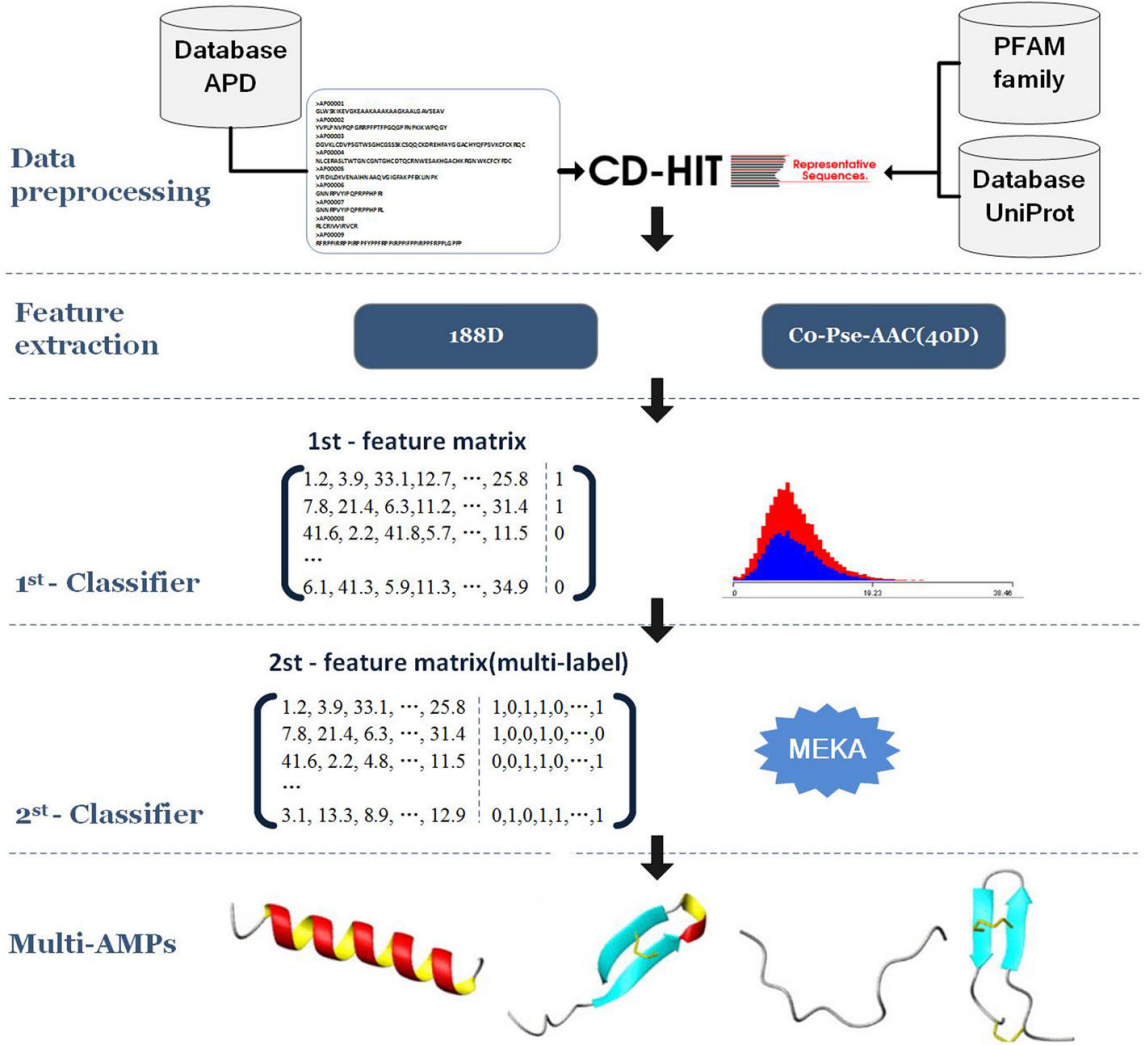
The main flowchart of the AMPs identification and prediction process

Firstly, we extracted SVM-prot 188-D features and Co-Pse-AAC 40-D features for each peptide sequence. Then the first-layer classifier was followed for identifying if the sequence is AMPs or not. Several common classifiers, including Random Forest (RF), Bagging, J48, OneR, Naive Bayesian NB, KNN, and LibSVM, were chosen for performance comparison. The result showed that the Random Forest and Bagging classifiers based on decision trees have achieved the highest prediction accuracy rate that exceeded 84% for both SVM-prot 188-D and Co-Pse-AAC 40-D features (Fig. 1).

We further applied 1660 test dataset samples *S*_*test*_ to verify 5 RF and Bagging based classifiers (188D-RF–W, 188D-RF–R, 188D-Bagging–W, 188D-Bagging–R, 40D-RF-R), where W denotes weighted random sampling, and R denotes random-under-sampling, since the AMP dataset is highly imbalanced, whereas sampling methods might affect the prediction performance significantly.

Table 3 shows that the **188D-RF-W** classifier based on weighted random sampling can guarantee good sensitivity and specificity on both training set and test set, which can efficiently identify AMPs and non-AMPs, where TPR represents true positive rate, FPR represents false positive rate, and AUC is area under the curve. Hence, we use it as the first-layer classifier of our proposed MAMP-Pred method. FPR TPR AUC

**Table 3:** Performance comparison of first-layer classifiers on test dataset *S*_*test*_

Classifier	AMPs			non-AMPs			Acc(%)
	TPR	FPR	AUC	TPR	FPR	AUC	
**188D-RF-W**	**0.831**	**0.156**	**0.900**	**0.844**	**0.169**	**0.900**	**84.157**
188D-RF-R	0.892	0.205	0.897	0.795	0.108	0.897	81.145
188D-Bagging-W	0.888	0.205	0.899	0.795	0.112	0.899	81.084
188D-Bagging-R	0.921	0.220	0.897	0.780	0.079	0.897	80.361
40D-RF-R	0.874	0.194	0.890	0.806	0.126	0.890	81.747

a. Statements that serve as captions for the entire table do not need footnote letters

b. W = weighted random sampling, R = random-under-sampling, 188D = SVM-prot 188-D, 40D = Co-Pse-AAC 40-D

### Second classifier - Identifying function types of AMPs

We investigated several multi-label classification methods on dataset *s*^*AMPs*^ in order to find the best classifier for identifying AMPs function types. We firstly evaluated different problem transformation methods, including Binary Correlation (BR), Classifier Chain (CC), Bayesian Classifier Chain (BCC), Tag Combination (LC), pruning set (PS), combined with representative single-label classifiers including J48, Random Tree, Random Forest, KNN and Bagging. We also investigated several adapted algorithm methods such as MLkNN, BRkNN, BP neural network, BPMLL, and DeepML, whereas the details were not illustrated in this paper due to the space limitations.

All multi-label classifiers have adopted train/test dataset split and 10-fold cross-validation mechanisms based on *s*^*AMPs*^ for evaluation. The evaluation results of BR-RF, PS-RF, CC-RF, BCC-RF, LC-RF and BRkNN methods on dataset *s*^*AMPs*^ are shown in Table 4. It can be seen that **PS-RF** and **LC-RF** have achieved the highest overall accuracy, and 10-fold cross-validation performs better than train/test dataset split mechanism for all problem transformation methods.

**Table 4:** Performance Comparison of Second-layer Classifiers (10 fold cross-validation)

Models	Acc	EMR	H-Loss	F1-Micro	F1-Macro	One-error	Rank-Loss	Log-Loss
BR-RF	0.839	0.785	0.021	0.920	0.941	0.122	0.019	0.076
**PS-RF**	**0.856**	**0.825**	**0.020**	**0.923**	**0.939**	**0.138**	**0.052**	**0.056**
CC-RF	0.844	0.794	0.021	0.922	0.942	0.165	0.051	0.057
BCC-RF	0.847	0.801	0.020	0.924	0.943	0.160	0.051	0.056
**LC-RF**	**0.855**	**0.824**	**0.020**	**0.923**	**0.939**	**0.139**	**0.052**	**0.056**
BRkNN	0.696	0.561	0.044	0.838	0.783	0.238	0.101	0.121

**Fig. 2:**
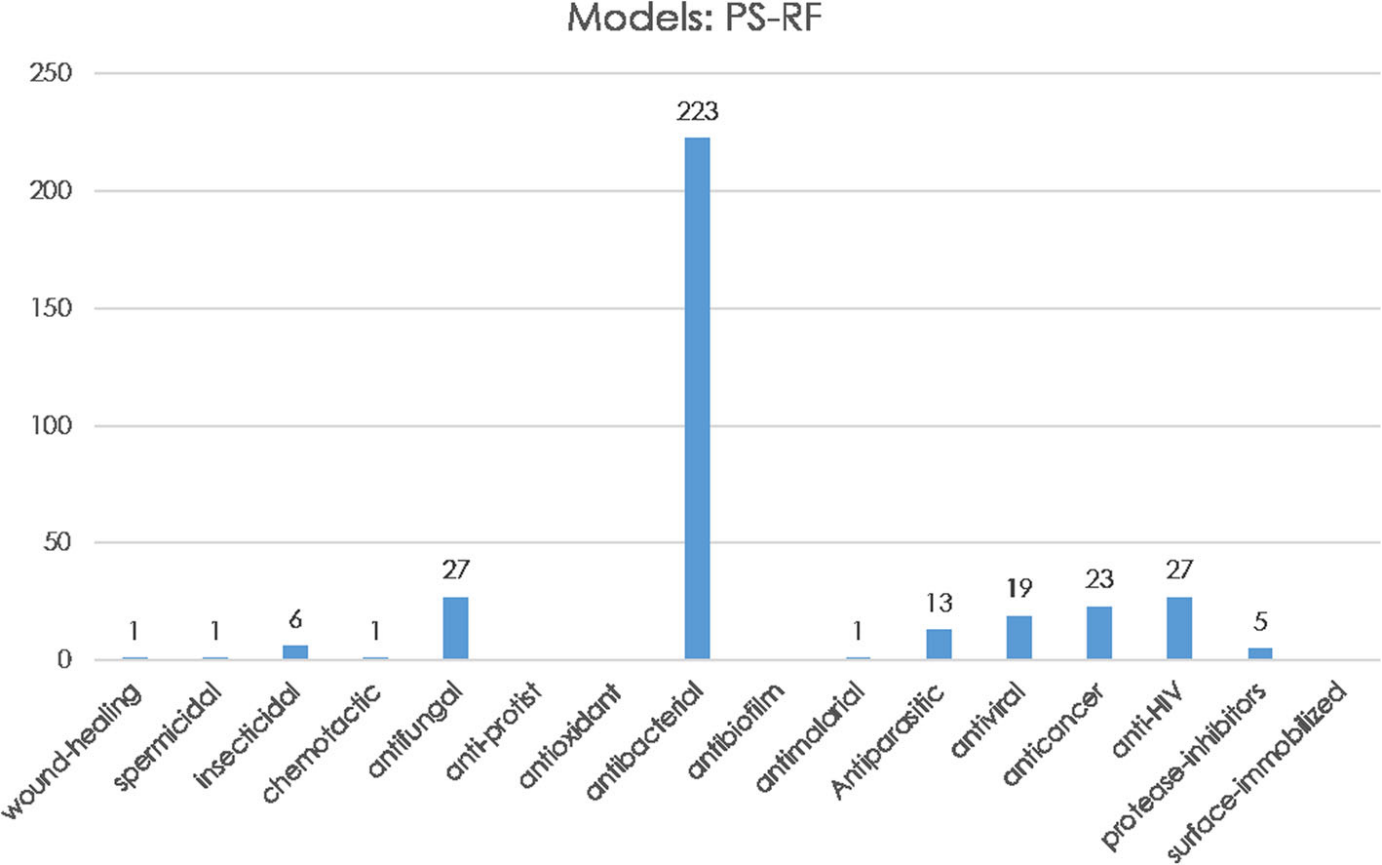
Predicting function types of *s*^*non*−*AMPs*−*NOACT*^

The second stage is to apply PS-RF and LC-RF classifiers for predicting the possible antimicrobial activities or function types of the 278 AMPs with unknown antibacterial activity *s*^*non*−*AMPs*−*NOACT*^. Similar prediction results were obtained in PS-RF and LC-RF. As shown in Fig. 2, there is one wound healing activity, one spermicidal activity, one chemotactic activity, one antimalarial activity, 6 Insecticidal activities, 27 antifungal activities, 27 anti-HIV activities, 13 Antiparasital activities, 19 antiviral activities, 23 anticancer activities, 5 proteinase inhibitor activities, 223 antibacterial activities. In addition, none of the antimicrobial peptides may have anti-protist, antioxidant, antibiotics, and surface immobilized activities.

### Performance evaluation

To benchmark our method, we present a comparative analysis of our MAMPs-Pred method against other existing best-performing in literature. Most of the existing methods can only be used to identify a query peptide as an AMP or non-AMP.

To make the comparison feasible and applicable, we firstly compared the first-layer classifier of MAMPs-Pred with the first-level classifier of iAMP-2L. We have applied the independent test data sets $S_{test}^{Ind}$ in [9], which contains 920 AMPs and non-AMPs sequences. The overall accuracy rate of iAMP-2L was 86.32%. Our mechanism has achieved 87.14% classification accuracy, which shows better performance than iAMP-2L, as shown in Table 5.

**Table 5:** Performance comparison of MAMPs-Pred and iAMP-2L first-layer on $S_{test}^{Ind}$ dataset)

Method	Acc	SN	SP	Mcc
MAMPs-Pred	93.91%	92.83%	94.99%	0.878
iAMP-2L	92.23%	97.72%	86.74%	0.845

The second-layer classifier of MAMPs-Pred was compared with the iAMP-2L method [9] and LIFT classification method proposed in [17]. It can be seen that our MAMPs-Pred method has gained an improved overall performance over iAMP-2L and LIFT as shown in Table 6.

**Table 6:** Performance comparison of MAMPs-Pred and iAMP-2L, LIFT second-layer on $S_{test}^{Ind}$ data set

Method	Acc	EMR	Precision	Recall	H-Loss
MAMPs-Pred	0.856	0.825	0.918	0.929	0.020
iAMP-2L	0.669	0.43	0.833	0.75	0.164
LIFT	0.700	0.5365	0.838	0.741	0.1392

The first reason is that the amino acid composition and its eight physicochemical properties which are used for feature extraction in this study, can better express the relationship between structure and antimicrobial peptides function types thus yield significantly improved performance.

The second reason is that the pruning set method applied in the second-layer multi-label classification, which transforms the label set into a single label in the problem, and directly models the label correlation, can achieves an overall better prediction performance.

### Performance on predicting Penaeus AMPs

In total 14298 protein sequences of shrimp (Penaeus) were fetched from the public UniProt database, including Penaeus monodon, Penaeus vannamei, etc. We then obtained 1452 sequences with a length between 5 and 100 from the 14298 sequences, followed by extracting SVM-prot 188-D features based on amino acid composition (AAC) and its 8 physicochemical properties for each penaeus protein sequence. The processed sequences were subsequently fed to the first-layer classifier of MAMP-Pred. A total of 126 AMPS/AMPS-like sequences were detected, accounting for 8.68*%* of the total sequence.

**Fig. 3:**
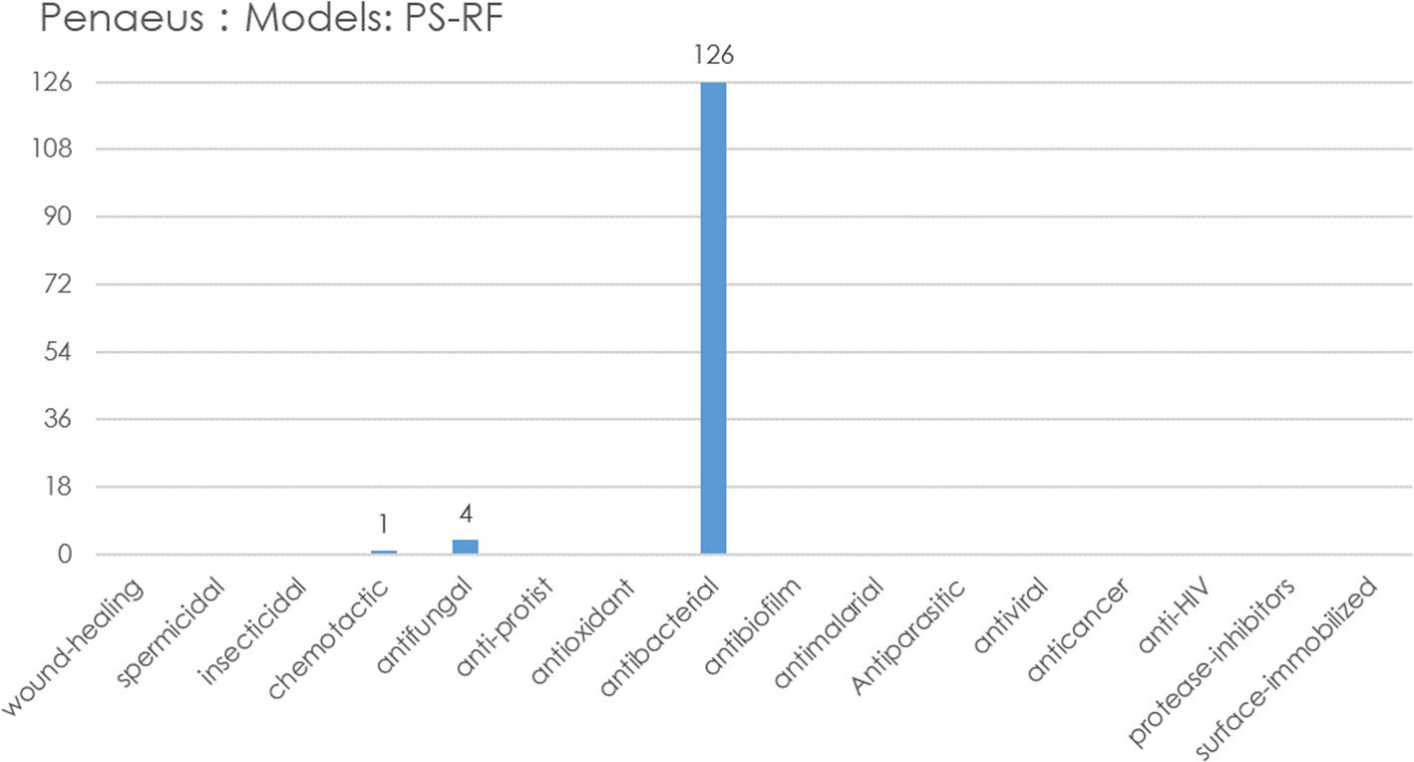
AMPs activity prediction of 126 shrimp sequences

In the second-layer multi-label classification, we have predicted the possible antimicrobial activities or function types that an AMP belongs to. All 126 penaeus AMPs sequences had antibacterial activity, one with chemotactic activity, and four with antifungal activity, as shown in Fig. 3. MAMP-Pred can be regarded as an efficient data-mining method to predict the potential antimicrobial peptides and antibacterial activities of the query sequences.

## Discussion

Antimicrobial peptides are increasingly gaining considerable attention both from research and industry, as well as clinical interest. With the growing microbial resistance to conventional antimicrobial agents, the demand for unconventional and efficient AMPs has become urgent. Effective usage of AMPs and their derivatives can greatly improve the immunity and breeding survival rate of aquatic products.

The results reported in this study indicate that the MAMP-Pred method achieves high performance for identifying AMPs and its functional types. The proposed approach is believed to supplement the tools and techniques that have been developed in the past for prediction of AMPs. The primary reason is that the amino acid composition and its eight physicochemical properties which are used for the feature extraction in this study, can better express the relationship between structure and antimicrobial peptides function types. The second reason is that the pruning set method applied in the second-layer multi-label classification achieves an overall higher prediction performance.

As summarized in [41], the recognition accuracy of machine learning methods ranges from the upper 70 to the lower 90 percent. Reported recognition accuracy has steadily improved over the past decade, while there is room for improvement.

The current MAMP-Pred approach can be straightforwardly extended in following directions in future research work:

1. Construct a more reliable datasets of positive and negative samples to reduce potential bias of model training introduced by sequence homology. We also believe that with more data available in the future, the prediction accuracy can be significantly enhanced.

2. The two-level prediction requires learning and classification to be performed twice, which lowers down the prediction efficiency. An adaptive dynamic approach which possibly yields faster speed and higher efficiency is of definite interest in our future research.

3. In this approach, the overlay of prediction errors might incur significant drop of prediction accuracy. In future work, the current method shall be straightforwardly extended to address these issues.

4. Predicting the AMPs and their function types of penaeus by this method can help us to understand the immune system of marine species. In addition, it eases subsequent mining and exploration of antimicrobial activity of other species. The predictor holds very high potential to become a useful high throughput tool to predict antimicrobial activity of other species.

## Conclusion

In this study, we made an attempt to develop an advanced machine learning based computational approach, MAMPs-Pred, for identification of AMPs and its function types. Initially, SVM-prot 188-D features were extracted that were subsequently used as input to a two-layer multi-label classifier. The first layer is to identify whether it is an AMP by applying RF classifier, and the second layer addresses the multitype problem by identifying the activities or function types of AMPs by applying PS-RF and LC-RF classifiers.

## Abbreviations

Acc: Overall accuracy
AMPs: Antimicrobial peptides
APD: Antimicrobial peptide database
CD-HIT: Cluster database at high identity with tolerance
ClassAMP: A method to predict the propensity of a peptide sequence
Co-Pse-AAC: Pseudo amino acid composition
EMR: Exact-match ratio
FKNN: Fuzzy K-nearest neighbour
H-Loss: Hamming-loss
iAMP-2L: A two-level multilabel classifier
LC-RF: Label combination-random forests
LIFT: Zhou’s multi-label learning algorithm
MAMPs-Pred: Our method
Mcc: Matthew’s correlation coefficient
PS-RF: Pruned sets-random forests
RF: Random forests
HMMS: Hidden Markov models
SN: Sensitivity
SP: Specificity
SVM-prot 188-D: A web server for protein classification with 188-D feature
SVM: Support vector machine

## References

[1] Malmsten M (2014). Antimicrobial peptides. Ups J Med Sci.

[2] Torrent Marc, Victoria Nogues M., Boix Ester (2012). Discovering New In Silico Tools for Antimicrobial Peptide Prediction. Current Drug Targets.

[3] Nannette YY, Michael RY (2004). Multidimensional signatures in antimicrobial peptides. Proc Natl Acad Sci.

[4] Meher PK, Sahu TK, Saini V, Rao AQ. Predicting antimicrobial peptides with improved accuracy by incorporating the compositional, physico-chemical and structural features into chou’s general PseAAC; 2017. 10.1038/srep42362. PMC5304217 28205576

[5] Khosravian M (2013). Predicting antibacterial peptides by the concept of chou’s pseudo-amino acid composition and machine learning methods. Protein Pept Lett.

[6] Niarchou Anastasia, Alexandridou Anastasia, Athanasiadis Emmanouil, Spyrou George (2013). C-PAmP: Large Scale Analysis and Database Construction Containing High Scoring Computationally Predicted Antimicrobial Peptides for All the Available Plant Species. PLoS ONE.

[7] Lin H. H., Han L. Y., Cai C. Z., Ji Z. L., Chen Y. Z. (2005). Prediction of transporter family from protein sequence by support vector machine approach. Proteins: Structure, Function, and Bioinformatics.

[8] Wang Ping, Hu Lele, Liu Guiyou, Jiang Nan, Chen Xiaoyun, Xu Jianyong, Zheng Wen, Li Li, Tan Ming, Chen Zugen, Song Hui, Cai Yu-Dong, Chou Kuo-Chen (2011). Prediction of Antimicrobial Peptides Based on Sequence Alignment and Feature Selection Methods. PLoS ONE.

[9] Xiao Xuan, Wang Pu, Lin Wei-Zhong, Jia Jian-Hua, Chou Kuo-Chen (2013). iAMP-2L: A two-level multi-label classifier for identifying antimicrobial peptides and their functional types. Analytical Biochemistry.

[10] Joseph Shaini, Karnik Shreyas, Nilawe Pravin, Jayaraman V. K., Idicula-Thomas Susan (2012). ClassAMP: A Prediction Tool for Classification of Antimicrobial Peptides. IEEE/ACM Transactions on Computational Biology and Bioinformatics.

[11] Lira Felipe, Perez Pedro S., Baranauskas José A., Nozawa Sérgio R. (2013). Prediction of Antimicrobial Activity of Synthetic Peptides by a Decision Tree Model. Applied and Environmental Microbiology.

[12] Fjell Christopher D., Hancock Robert E.W., Cherkasov Artem (2007). AMPer: a database and an automated discovery tool for antimicrobial peptides. Bioinformatics.

[13] Veltri Daniel, Kamath Uday, Shehu Amarda (2018). Deep learning improves antimicrobial peptide recognition. Bioinformatics.

[14] Schneider Petra, Müller Alex T., Gabernet Gisela, Button Alexander L., Posselt Gernot, Wessler Silja, Hiss Jan A., Schneider Gisbert (2016). Hybrid Network Model for “Deep Learning” of Chemical Data: Application to Antimicrobial Peptides. Molecular Informatics.

[15] Wang Z, Wang G (2004). APD: the antimicrobial peptide database. Nucleic Acids Res.

[16] Wang G (2009). Li, Wang Z. APD2: the updated antimicrobial peptide database and its application in peptide design. Nucleic Acids Res.

[17] Wang Pu, Xiao Xuan (2013). Multi-Label Classifier Design for Predicting the Functional Types of Antimicrobial Peptides. Advanced Materials Research.

[18] Zhou HL (2014). A Multi-label classifier for prediction membrane protein functional types in animal. J Membr Biol.

[19] Cai C.Z. (2003). SVM-Prot: web-based support vector machine software for functional classification of a protein from its primary sequence. Nucleic Acids Research.

[20] Li Ying Hong, Xu Jing Yu, Tao Lin, Li Xiao Feng, Li Shuang, Zeng Xian, Chen Shang Ying, Zhang Peng, Qin Chu, Zhang Cheng, Chen Zhe, Zhu Feng, Chen Yu Zong (2016). SVM-Prot 2016: A Web-Server for Machine Learning Prediction of Protein Functional Families from Sequence Irrespective of Similarity. PLOS ONE.

[21] Huang Ying, Niu Beifang, Gao Ying, Fu Limin, Li Weizhong (2010). CD-HIT Suite: a web server for clustering and comparing biological sequences. Bioinformatics.

[22] Zou Quan, Wang Zhen, Guan Xinjun, Liu Bin, Wu Yunfeng, Lin Ziyu (2013). An Approach for Identifying Cytokines Based on a Novel Ensemble Classifier. BioMed Research International.

[23] Zeng XX (2015). Identification of cytokine via an improved genetic algorithm. Front Comput Sci.

[24] Cheng Xian-Ying, Huang Wei-Juan, Hu Shi-Chang, Zhang Hai-Lei, Wang Hao, Zhang Jing-Xian, Lin Hong-Huang, Chen Yu-Zong, Zou Quan, Ji Zhi-Liang (2012). A Global Characterization and Identification of Multifunctional Enzymes. PLoS ONE.

[25] Zou Q, Chen W, Huang Y, Liu X, Jiang Y (2013). Identifying multi-functional enzyme with hierarchical multi-label classifier. J Comput Theor Nanosci.

[26] Chou KC (2005). Using amphiphilic pseudo amino acid composition to predict enzyme subfamily classes. Bioinformatics.

[27] Bin L (2015). Pse-in-One: a web server for generating various modes of pseudo components of DNA, RNA, and protein sequences. Nucleic Acids Res.

[28] Song Li, Li Dapeng, Zeng Xiangxiang, Wu Yunfeng, Guo Li, Zou Quan (2014). nDNA-prot: identification of DNA-binding proteins based on unbalanced classification. BMC Bioinformatics.

[29] Zou Q, Guo M, Liu Y, Wang J (2010). A Classification method for class-imbalanced data and its application on bioinformatics. J Comput Res Dev.

[30] Lin S (2011). Under-sampling method research in class-imbalanced data. J Comput Res Dev.

[31] Batista GE, Prati RC, Monard MC (2004). A study of the behavior of several methods for balancing machine learning training data. ACM Sigkdd Explor Newsl.

[32] Guo LJ (2013). Research on imbalanced data classification based on ensemble and under-sampling. J Front Comput Sci Technol.

[33] Tsoumakas G, Katakis I (2007). Multi label classification: an overview. Int J Data Warehous Min.

[34] Guo SH (2014). iNuc-PseKNC: a sequence-based predictor for predicting nucleosome positioning in genomes with pseudo k-tuple nucleotide composition. Bioinformatics.

[35] Lin H, Deng EZ, Ding H, Chen W, Chou KC (2014). iPro54-PseKNC: a sequence-based predictor for identifying sigma-54 promoters in prokaryote with pseudo k-tuple nucleotide composition. Nucleic Acids Res.

[36] Tang Hua, Chen Wei, Lin Hao (2016). Identification of immunoglobulins using Chou's pseudo amino acid composition with feature selection technique. Molecular BioSystems.

[37] Zhu PP (2015). Predicting the subcellular localization of mycobacterial proteins by incorporating the optimal tripeptides into the general form of pseudo amino acid composition. Mol Biosyst.

[38] Chen W, Feng P, Ding H, Lin H, Chou KC (2015). iRNA-Methyl: Identifying N(6)-methyladenosine sites using pseudo nucleotide composition. Anal Biochem.

[39] Chen Wei, Feng Pengmian, Lin Hao (2012). Prediction of replication origins by calculating DNA structural properties. FEBS Letters.

[40] Chen Wei, Feng Peng-Mian, Lin Hao, Chou Kuo-Chen (2014). iSS-PseDNC: Identifying Splicing Sites Using Pseudo Dinucleotide Composition. BioMed Research International.

[41] Veltri Daniel, Kamath Uday, Shehu Amarda (2017). Improving Recognition of Antimicrobial Peptides and Target Selectivity through Machine Learning and Genetic Programming. IEEE/ACM Transactions on Computational Biology and Bioinformatics.

